# Crystal engineering in all its hues in **IUCrJ**

**DOI:** 10.1107/S2052252520001608

**Published:** 2020-02-29

**Authors:** Gautam R. Desiraju

**Affiliations:** a Solid State and Structural Chemistry Unit, Indian Institute of Science, Bangalore 560 012, India

**Keywords:** crystal engineering, crystals, crystallography, editorial

## Abstract

The articles in crystal engineering and structural chemistry in **IUCrJ** published from 2019 onwards reflect the breadth and outreach of this new and important subject.

As the subject of crystal engineering diversifies and extends its outreach into many aspects of structural science, it is but natural that the 30 or so papers published in this area in **IUCrJ** during 2019 and early 2020 exemplify these trends. Crystal engineering is all about the design of molecular crystal structures and there is now greater emphasis on property design.

The present focus on pharmaceutical solids and the importance of crystal engineering in the synthesis of new solid forms, be they cocrystals, polymorphs or solvates, has resulted in a concomitant increase in papers dealing with such topics. Cocrystals are especially attractive from the design viewpoint: there is nascent interest in formulation technologies for cocrystals and one even comes across the term ‘cocrystal engineering’!

The 30 above-mentioned crystal engineering papers appear among the 120 or so that have been published in **IUCrJ** since the start of 2019. For comparison, about 150 papers in the crystal engineering area have been published since the inception of the journal itself. Recent papers that have been published in **IUCrJ** cover a variety of frontier topics and some representative papers are listed below (Alex *et al.*, 2019 ▸; Brink & Helliwell, 2019 ▸; Broadhurst *et al.*, 2020 ▸; Fatima *et al.*, 2020 ▸; Gianopoulos *et al.*, 2019 ▸; Oburn *et al.*, 2019 ▸; Ranjan *et al.*, 2020 ▸). These cover areas like charge density, polymorphism and isomorphism, mechanical properties, pharmaceutical solids, and core papers on supramolecular synthons and synthetic strategy. There is a keen interest in following crystallization mechanisms through an observation of essentially ‘static’ crystal structures and one can sense an impending and increasing interest in time-resolved and space-resolved crystallography towards these ends.

I would certainly like to see more papers in this section in 2020–2021, possibly more in the MOF/COF areas, and an increase in the journal impact factor. It is not easy to publish in **IUCrJ** and it is important to maintain this high benchmarking. The 24th Congress of the IUCr will take place this year and the program is, as usual, strong in crystal engineering and structural chemistry; it is to be hoped that this will have its impact on the number and quality of crystal engineering papers in **IUCrJ** during the coming year.
